# Correction: The chemical chaperone 4-phenylbutyric acid rescues molecular cell defects of *COL3A1* mutations that cause vascular Ehlers Danlos Syndrome

**DOI:** 10.1038/s41420-025-02529-2

**Published:** 2025-05-23

**Authors:** Ramla Omar, Michelle AW Lee, Laura Gonzalez-Trueba, Cameron R. Thomson, Uwe Hansen, Spyridonas Lianos, Snoopy Hazarika, Omar HMEH El Abdallah, Malak A. Ammar, Jennifer Cassels, Alison M. Michie, Neil J. Bulleid, Fransiska Malfait, Tom Van Agtmael

**Affiliations:** 1 School of Cardiovascular and Metabolic Health, College of Medical, Veterinary & Life Sciences, University of Glasgow, Glasgow, G12 8QQ UK; 2 Institute of Musculoskeletal Medicine, University of Muenster, University Hospital Muenster, D-48149 Muenster, Germany; 3 Paul O’Gorman Leukaemia Research Centre, School of Cancer Studies, College of Medical, Veterinary & Life Sciences, University of Glasgow, Glasgow, UK; 4 School of Molecular Biosciences, College of Medical, Veterinary & Life Sciences, University of Glasgow, Glasgow, G12 8QQ UK; 5 Center for Medical Genetics, Ghent University Hospital and Department for Biomolecular Medicine, Ghent University, Corneel Heymanslaan 10, 9000 Ghent, Belgium

**Keywords:** Endoplasmic reticulum, Aneurysm, Mechanisms of disease, Connective tissue diseases, Genetics research

Correction to: *Cell Death Discovery* 10.1038/s41420-025-02476-y, published online 25 April 2025

In this article, the author name Omar El El Abdallah has been corrected to Omar HMEH El Abdallah.

The original article has been corrected.

