# Supplementary material for: The chemical chaperone 4-phenylbutyric acid rescues molecular cell defects of COL3A1 mutations that cause vascular Ehlers Danlos Syndrome
Source: Cell Death Discov. 2025 Apr 25;11:200. doi: 10.1038/s41420-025-02476-y (PMC12032211; doi:10.1038/s41420-025-02476-y)
Supplement: Supplementary file 1 — Supplemental Data [file 41420_2025_2476_MOESM1_ESM.pdf]

The chemical chaperone 4-phenylbutyric acid rescues molecular cell defects of *COL3A1* mutations that cause vascular Ehlers Danlos Syndrome.

Ramla Omar[1], Michelle Lee[1], Laura Gonzalez-Trueba[1], Cameron R Thomson[1], Uwe Hansen[2], Spyridonas Lianos[1], Snoopy Hazarika[1], Omar MEH El-Abdallah[1], Malak, A Ammar[1], Jennifer Cassels[2], Alison M. Michie[2], Neil J Bulleid[3], Fransiska Malfait[4], Tom Van Agtmael[1 *]

Supplemental Figures

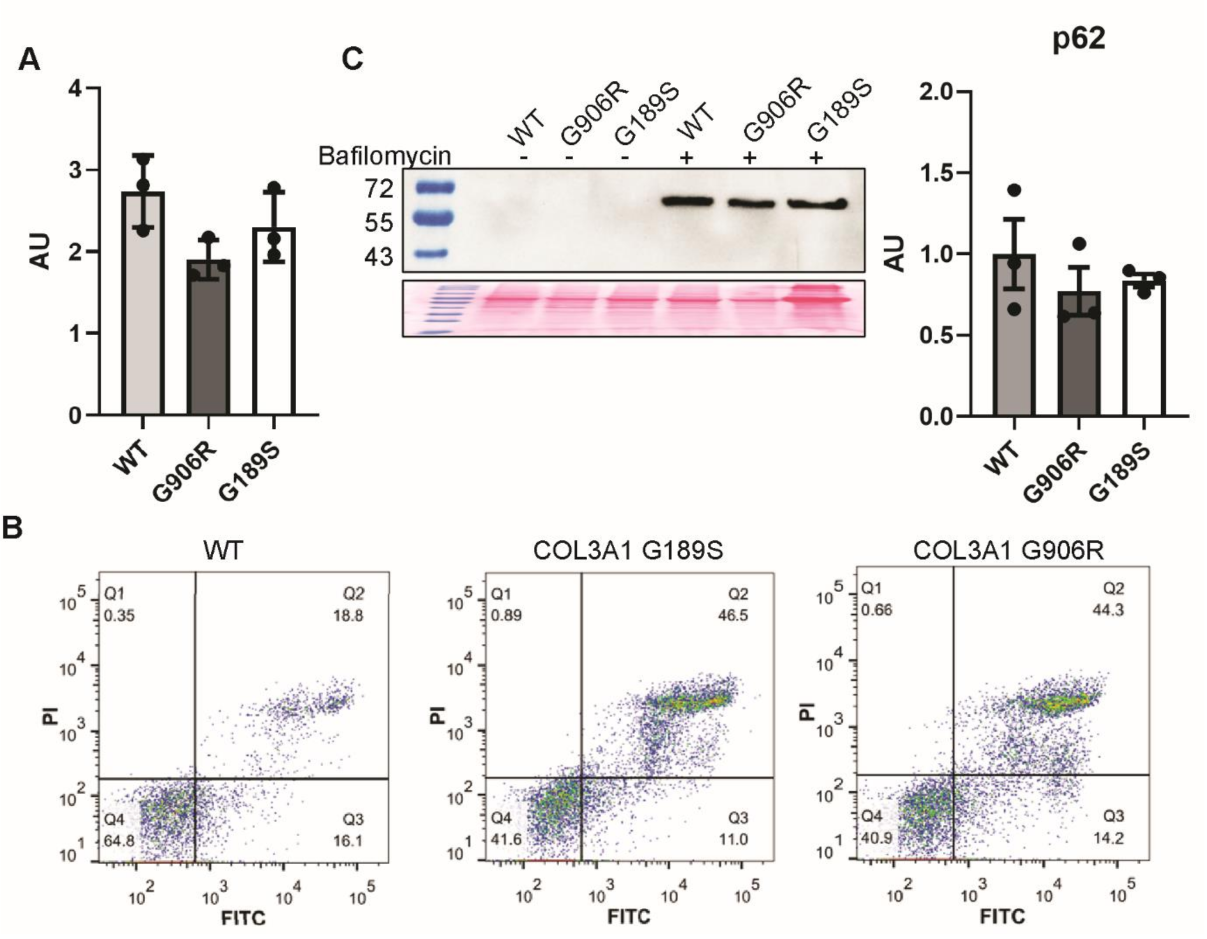

***Supplemental Figure 1**. (A) MTT assay* on wild type (WT) cells and *COL3A1*$^{G189S/+}$ (COL3A1 G189S) and *COL3A1*$^{G906R/+}$ (COL3A1 G906R) cells. Data represented as fold increase in signal between 0h and after 72 hours of cell culture (n=3, One Way ANOVA). *(B) COL3A1* mutations induce apoptosis. Scatter plot of FACS data shown in Figure 1 on wild type (WT) cells and *COL3A1*$^{G189S/+}$ (COL3A1 G189S) and *COL3A1*$^{G906R/+}$ (COL3A1 G906R) cells stained with propidium iodide (PI) and annexin V (FITC). (C) Western blotting against p62 on cells in the presence or absence of bafilomycin A1 (inhibitor or autohagic flux)(n=3, One Way ANOVA).

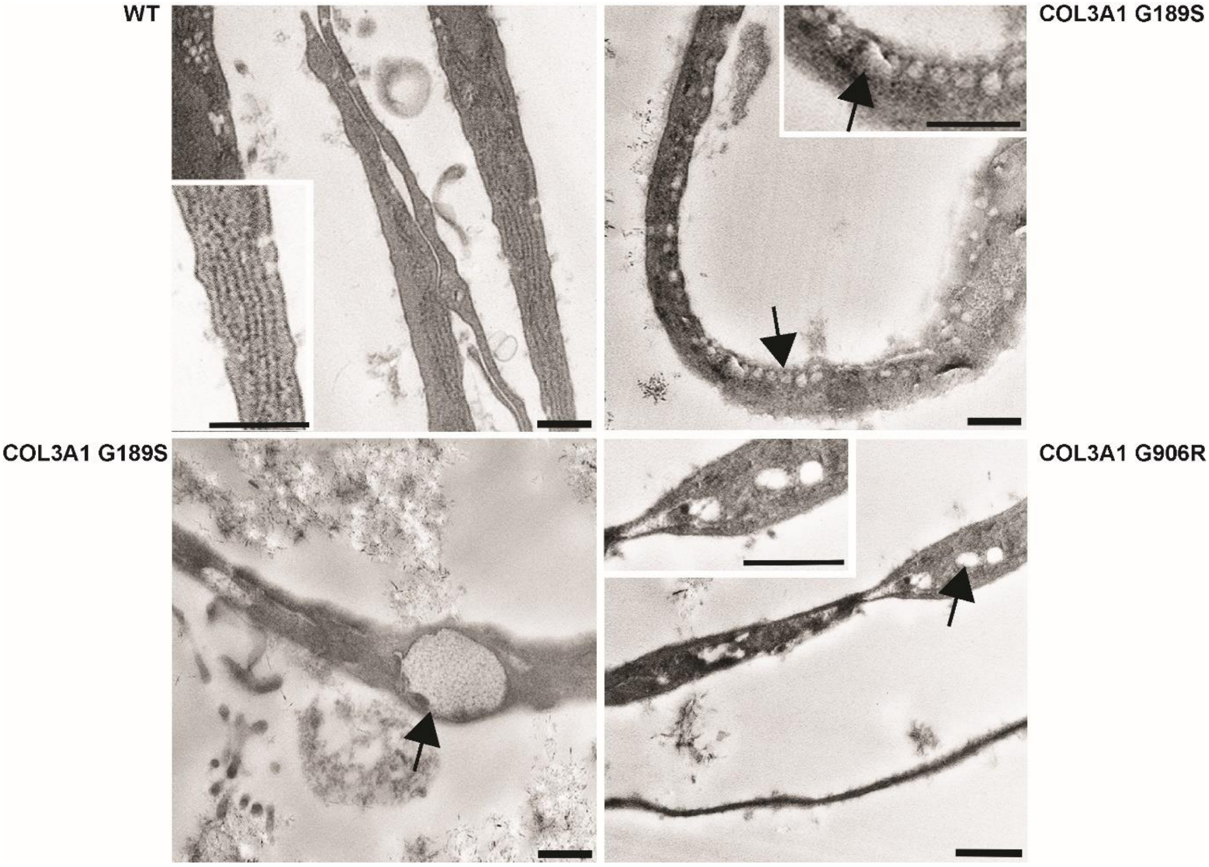

Supplemental Figure 2. EM analysis. TEM analysis on wild type (WT) cells and *COL3A1*$^{G189S/+}$ (COL3A1 G189S) and *COL3A1*$^{G906R/+}$ (COL3A1 G906R) cells. Swollen vesicles, indicating of distended ER, are indicated by black arrows. Size bar 500nm.

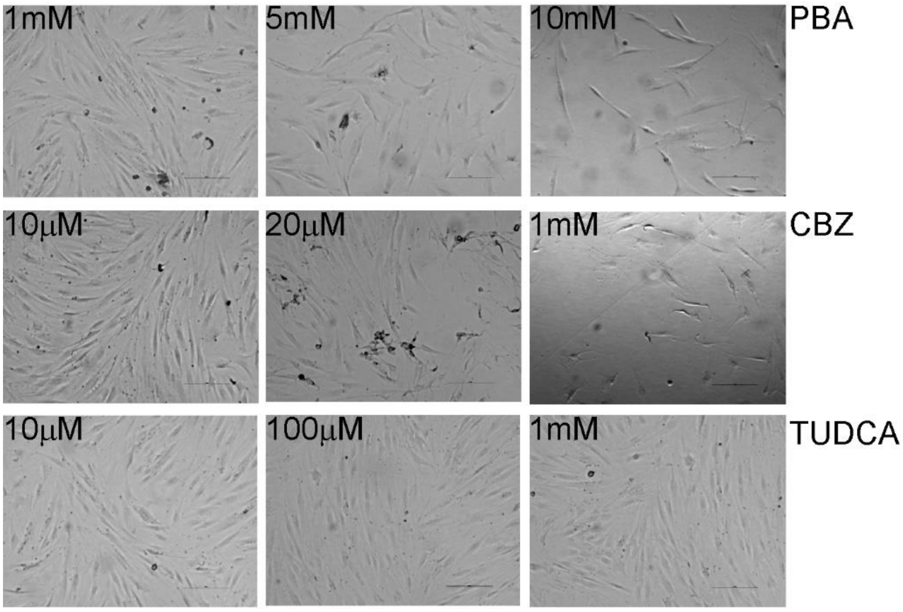

Supplemental Figure 3. Impact of treatments on cell viability. Phase contrast Microscopy images of wild type primary dermal fibroblasts incubated for 24 hours with varying concentrations of PBA, TUDCA and CBZ. Scale bar 100 µm

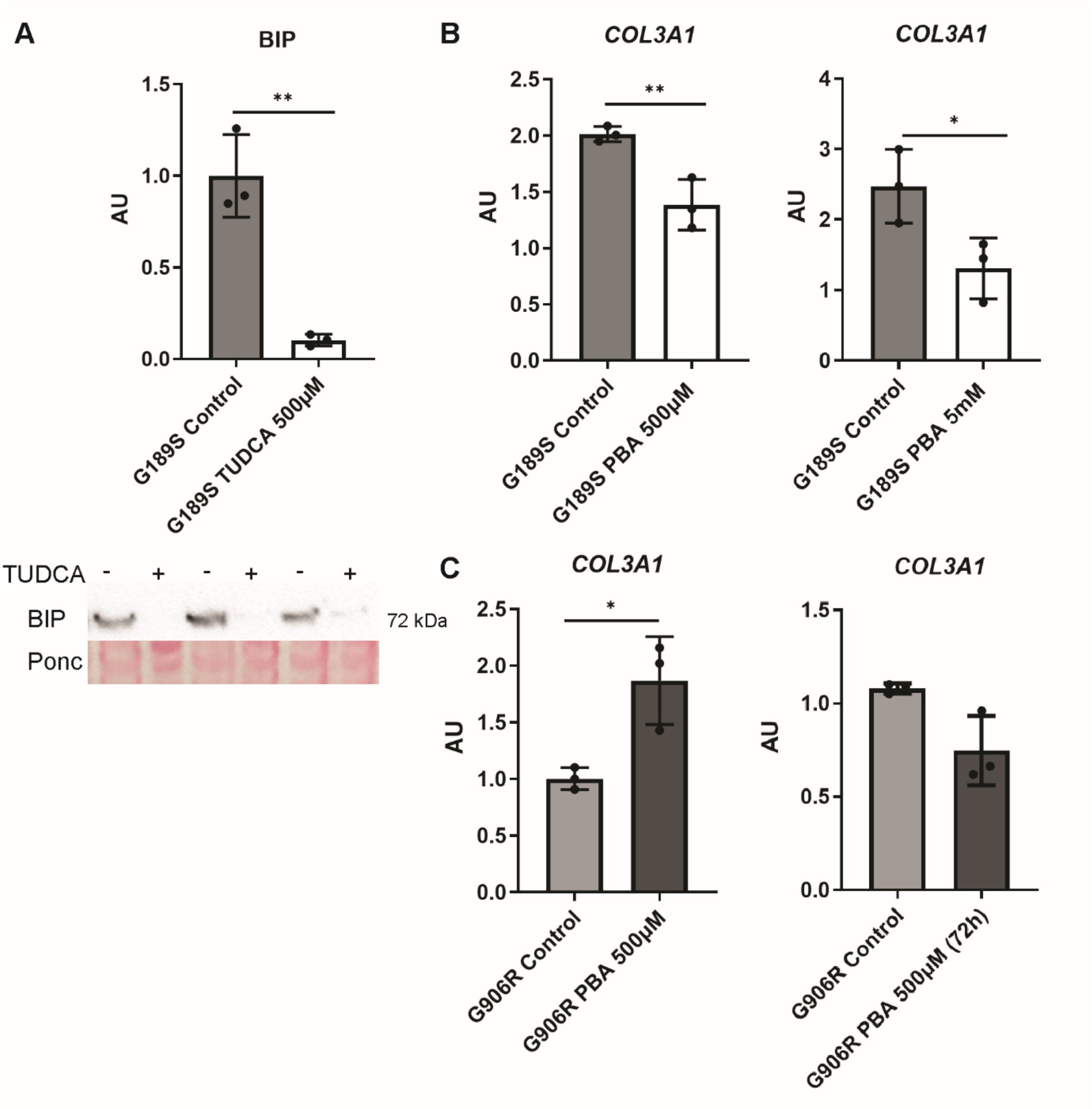

Supplemental Figure 4. Efficacy of TUDCA on ER stress and PBA on COL3A1 mRNA levels (A) *COL3A1*$^{G189S/+}$ (G189S) cells and cells treated with TUDCA for 24 hours, shows reduced levels of ER stress marker BIP. (B) *COL3A1*$^{G189S/+}$ (G189S) cells treated with 0.5 mM or 5mM PBA for 24 hours. (C) *COL3A1*$^{G906R/+}$ (G906R) cells treated with 0.5mM PBA for 24 or 72 hours. N=3, unpaired t-test *p<0.05, ** p<0.01

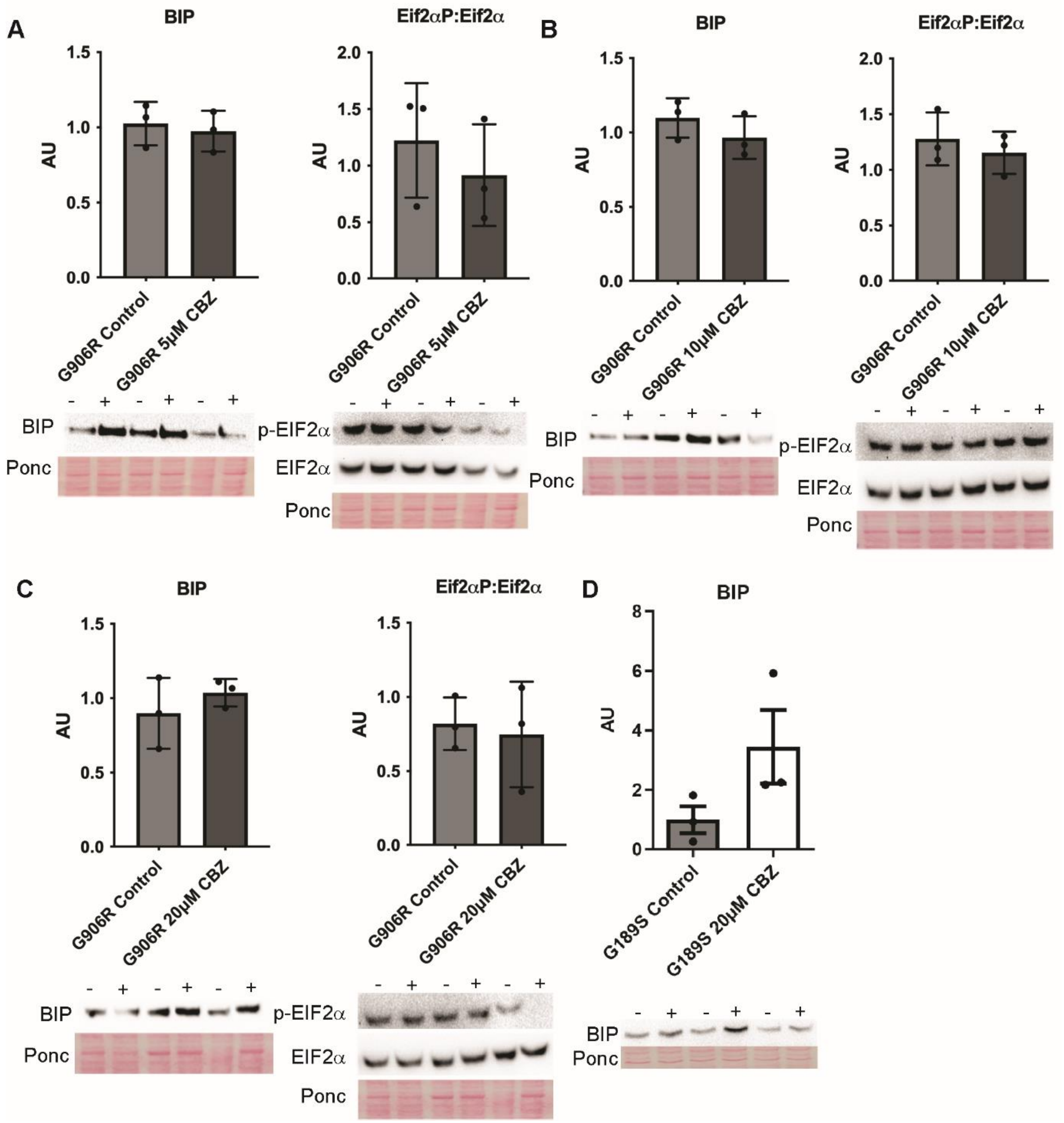

Supplemental Figure 5. CBZ Treatment does not reduce ER stress levels due to COL3A1 mutations. Cells were incubated for 24 hours with CBZ at different concentrations and western blotting was performed to assess protein levels of BIP, phosphorylated EIF2α (p-EIF2α) and total EIF2α as markers of ER stress. Ponceau (Ponc) used as protein loading control. (A) Western blot and graph of densitometry analysis of BIP and ratio of p-EIF2α:EIF2α in *COL3A1*$^{G906R/+}$ cells untreated (control) and incubated with 5 μM CBZ. A-D n =3, unpaired t-test (B) Data of incubation with *COL3A1*$^{G906R/+}$ cells untreated (control) and incubated with 10 μM CBZ. (C) Data of incubation with *COL3A1*$^{G906R/+}$ cells untreated (control) and incubated with 20 μM CBZ. (D) BIP protein levels in *COL3A1*$^{G189S/+}$ cells untreated (control) and incubated with 20 μM CBZ.

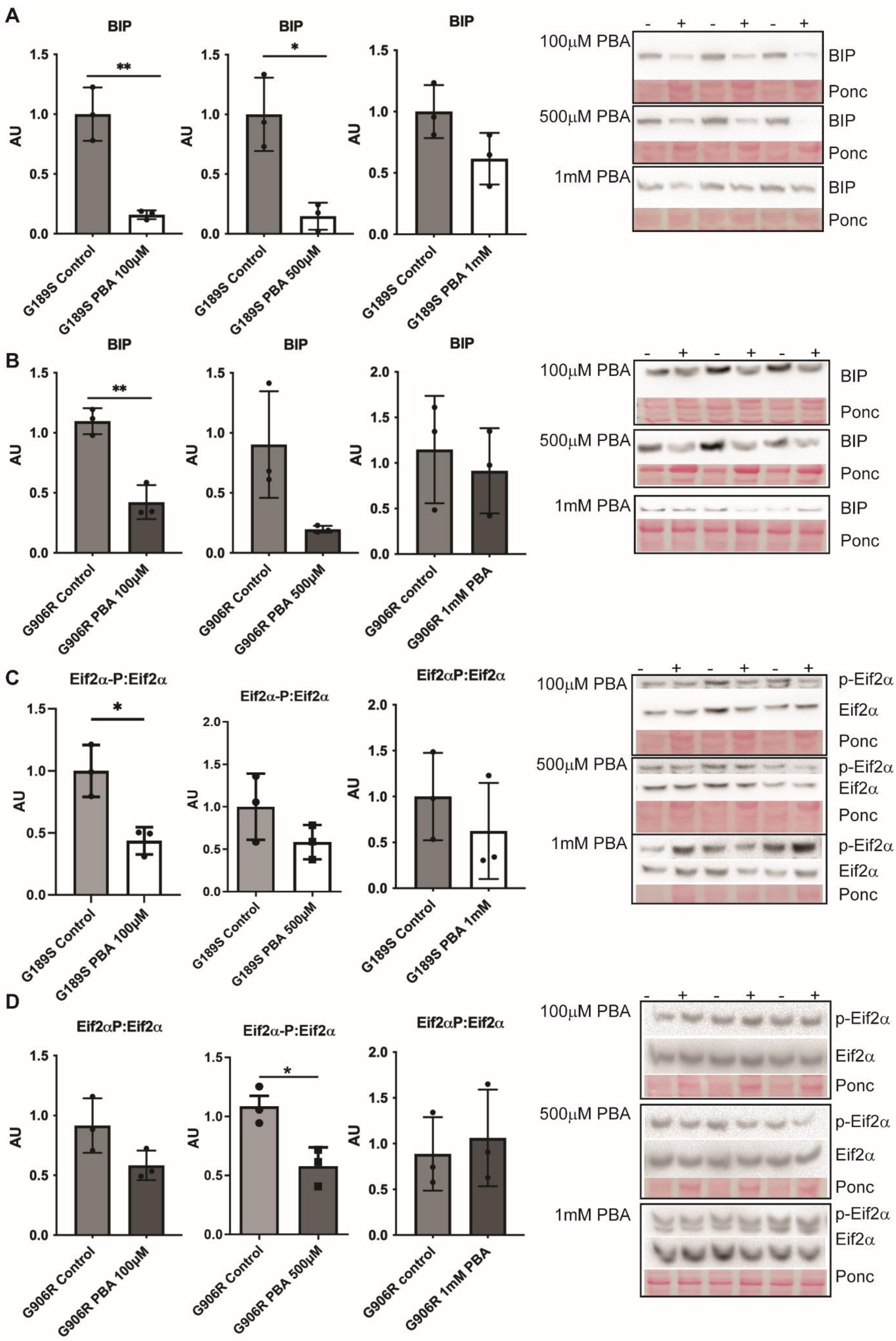
A
B
C
D
BIP
Eif2α-P:Eif2α
Eif2αP:Eif2α
AU
G189S Control
G189S PBA 100μM
G189S PBA 500μM
G189S PBA 1mM
G906R Control
G906R control
G906R PBA 100μM
G906R PBA 500μM
G906R 1mM PBA
100μM PBA
500μM PBA
1mM PBA
p-Eif2α
Eif2α
Ponc
**
*

Supplemental Figure 6. Dosage dependent effects of PBA. A graphic summary of these data is provided in Figure 4 C-D.(A) BIP protein levels in *COL3A1*$^{G189S/+}$ cells treated with PBA for 24 hours compared to untreated cells (control). Western blots are provided on right hand side. (B) BIP protein levels in *COL3A1*$^{G906R/+}$ cells treated with PBA for 24 hours compared to untreated cells (control). (C) Ratio of phosphorylated EIF2α (p-EIF2α): total EIF2α in *COL3A1*$^{G189S/+}$ cells treated with PBA compared to untreated cells. (D) Ratio of phosphorylated EIF2α (p-EIF2α): total EIF2α in *COL3A1*$^{G906R/+}$ cells treated with PBA compared to untreated cells. A-D n=3, unpaired t-test * $p<0.05$, ** $p<0.01$

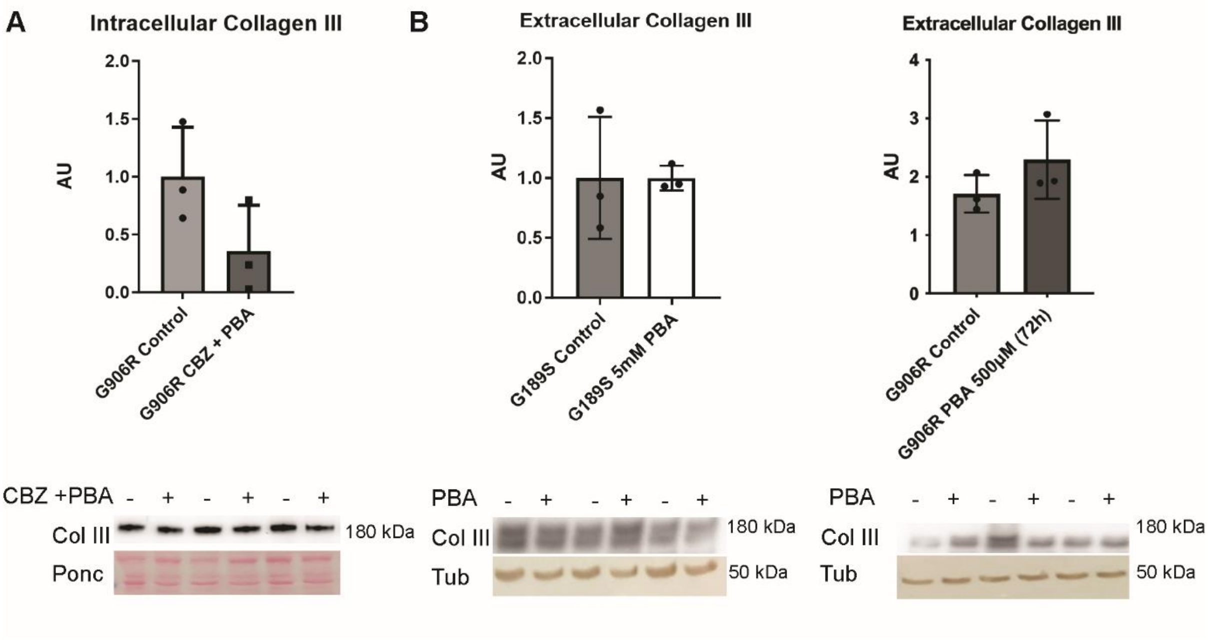

Supplemental Figure 7. Compound PBA:CBZ treatment and impact of PBA on collagen III secretion. (A) Western Blot against intracellular collagen III in COL3A1$^{G906R/+}$ cells treated (+) with combinatorial treatment of 500µM PBA and 20µM CBZ for 24 hours. Untreated cells (-, control). No reduction in intracellular collagen III was observed. (n=3, unpaired t-test) (B) Western Blot against collagen III on conditioned media of COL3A1$^{G189S/+}$ cells and COL3A1$^{G906R/+}$ treated (+) with PBA for 24 and 72 hours respectively. Untreated cells (-). Media was changed for the final 24 hours so conditioned media reflected the amount of collagen III secretion over a 24 hour period. No difference in collagen III secretion was observed. (n=3, unpaired t-test).
