# Supplementary material for: The chemical chaperone 4-phenylbutyric acid rescues molecular cell defects of COL3A1 mutations that cause vascular Ehlers Danlos Syndrome
Source: Cell Death Discov. 2025 Apr 25;11:200. doi: 10.1038/s41420-025-02476-y (PMC12032211; doi:10.1038/s41420-025-02476-y)
Supplement: Supplementary file 2 — Uncropped Western blots [file 41420_2025_2476_MOESM2_ESM.docx]

**The chemical chaperone 4-phenylbutyric acid rescues molecular cell defects of *COL3A1* mutations that cause vascular Ehlers Danlos Syndrome.**

Ramla Omar^1^, Michelle Lee^1^, Laura Gonzalez-Trueba^1^, Cameron R Thomson^1^, Uwe Hansen^2^, Spyridonas Lianos^1^, Snoopy Hazarika^1^, Omar MEH El-Abdallah^1^, Malak, A Ammar^1^, Jennifer Cassels^2^, Alison M. Michie^2^, Neil J Bulleid^3^, Fransiska Malfait^4^, Tom Van Agtmael^1 *^


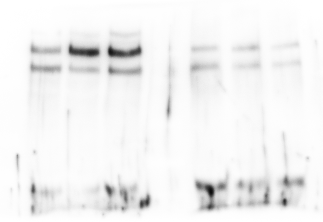
**Uncropped western blots**

Figure 2A intracellular (lanes 1-3) and extracellular (lanes 5-7) collagen III (Top band)


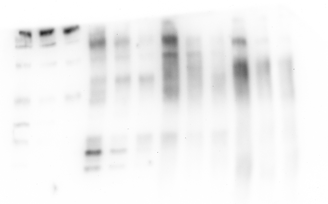


Figure 2D collagen III western trypsin


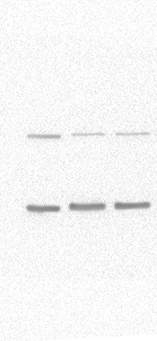

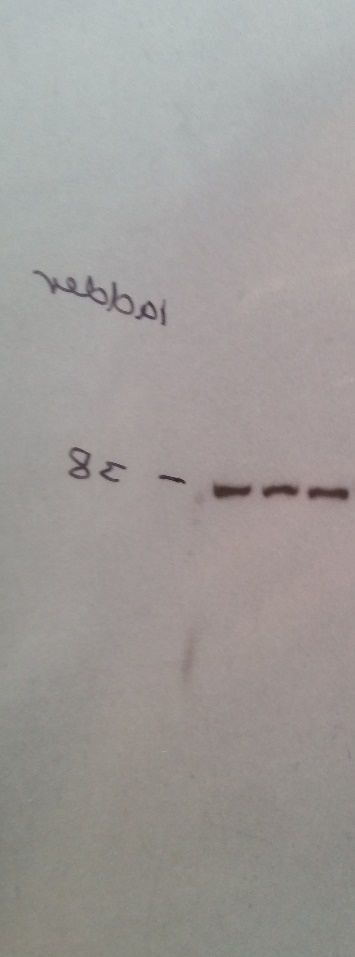

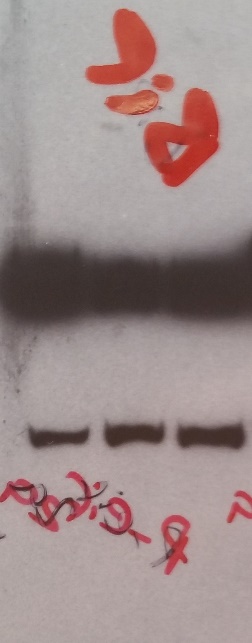

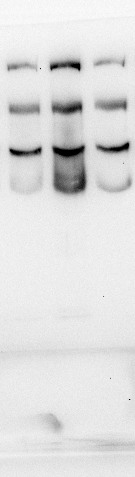


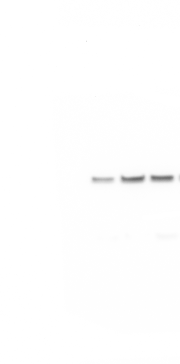


Figure 3A From left to right : Bip (left) and ATF4 (right, bottom band), P-eif2α (bottom band), total eif2α, ATF6 (top band)


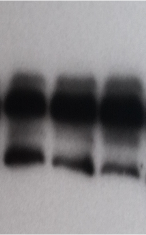

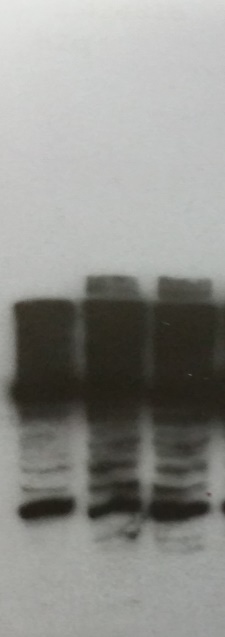


Figure 3E ubiquitin (left) and LC3 (right)


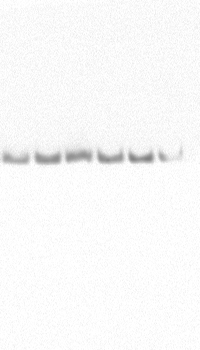

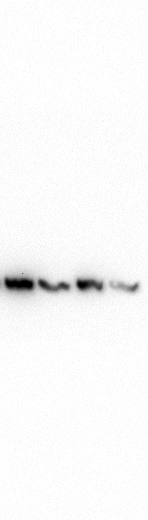


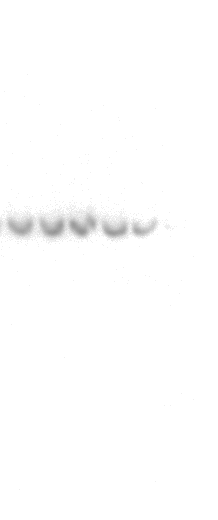

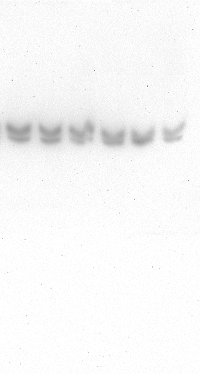
Figure 4A BIP G189S (left) and G906R (right)


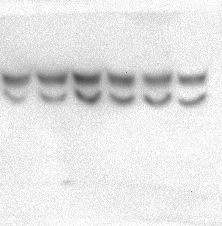

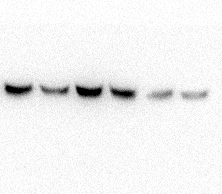


Figure 4B from left to right: G189S p-eif2Fα, G189S total eif2α, G906R p-eif2Fα, G906R total eif2α. Lanes 1-4 are shown in Figure 4B.

^
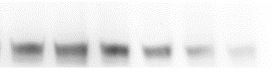
^
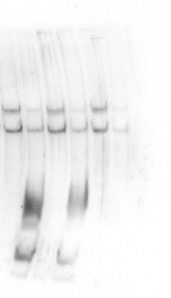

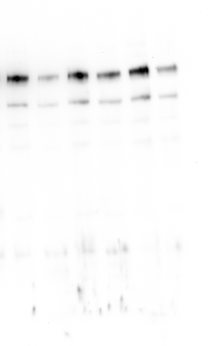


Figure 5A Collagen III from left to right: G189S (top band), G906R 24H, G906R 72H (top band)


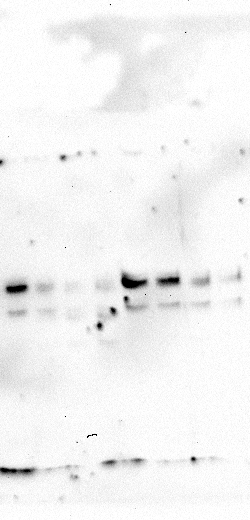

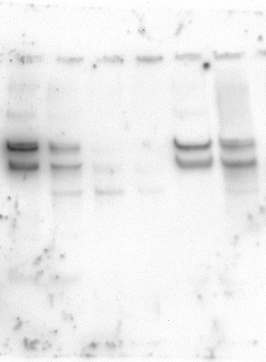


Figure 5E . Collagen III (top band of the 2 close bands) Left Gel : G189S. Right Gel G906R (left 4 lanes untreated with PBA, right 4 lanes treated with PBA)


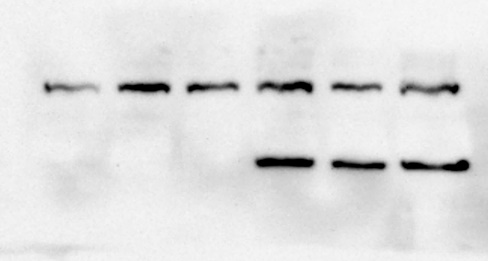


Supplemental Figure 1C p62


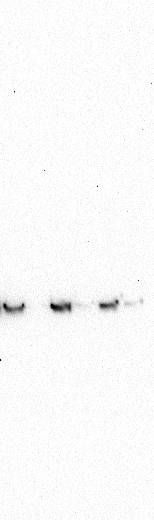


Supplemental Figure 3E Bip


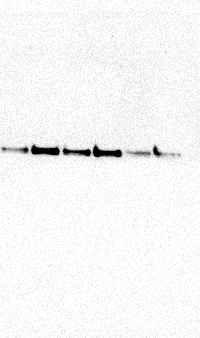


Supplemental Figure 4A BIP


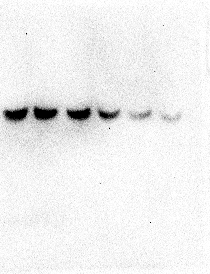


Supplemental Figure 4A p-Eif2α


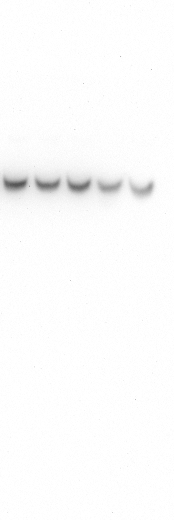


Supplemental Figure 4A Eif2α


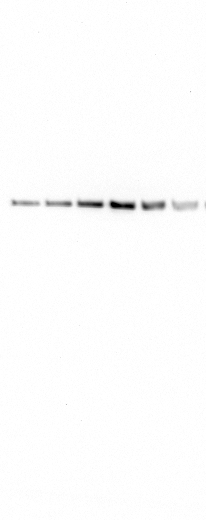


Supplemental Figure 4B BIP


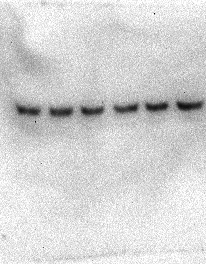


Supplemental Figure 4B p-Eif2α


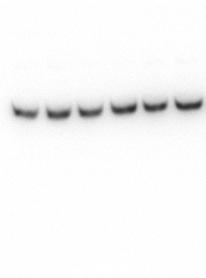


Supplemental Figure 4B Eif2α


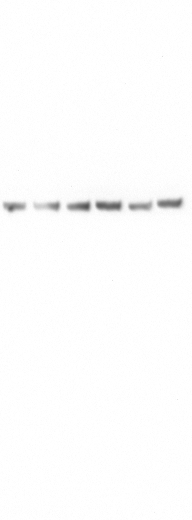


Supplemental Fig 4C Bip


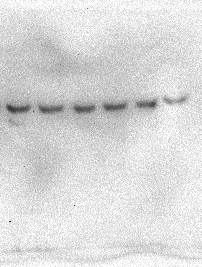


Supplemental Fig 4C p-Eif2α


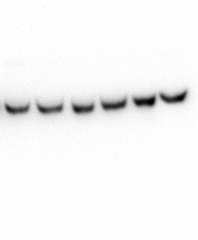


Supplemental Fig. 4C Eif2α


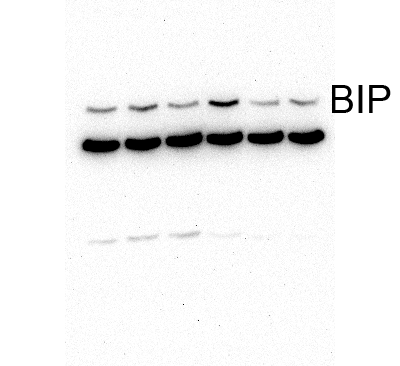


Supplemental Fig 4D BIP


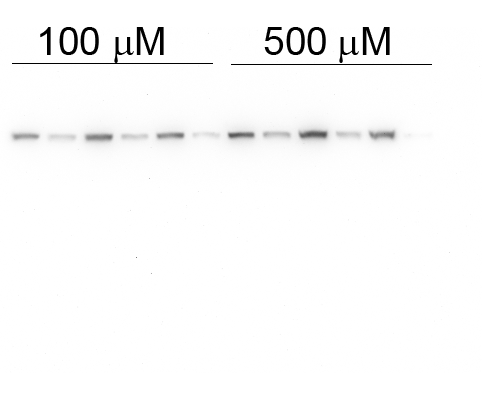


Supplemental Fig. 5A 100µM and 500µM PBA BIP


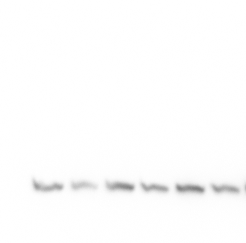


Supplemental Fig. 5A 1 mM PBA BIP


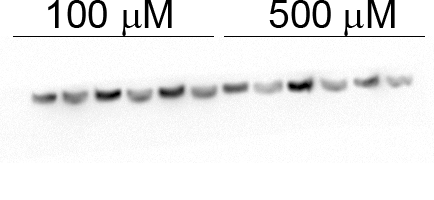


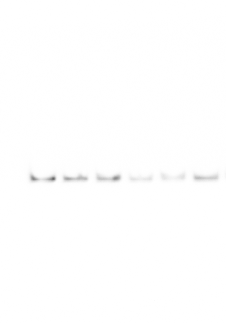
Supplemental Fig. 5B 100µM and 500µM PBA BIP

Supplemental Fig. 5B 1 mM PBA BIP


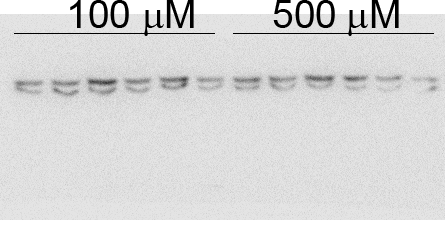


Supplemental Fig. 5C 100µM and 500µM PBA phospho-Eif2α


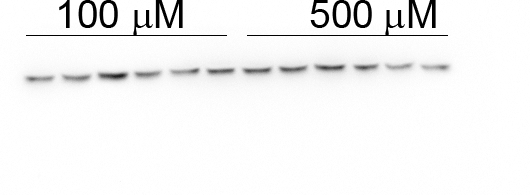


Supplemental Fig. 5C 100µM and 500µM PBA Eif2α


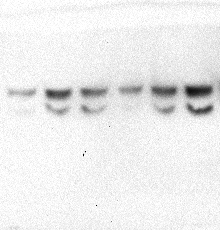


Supplemental Fig. 5C 1mM PBA phospho-Eif2α


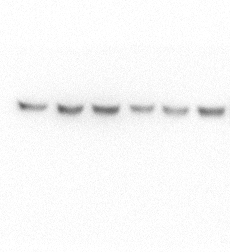


Supplemental Fig. 5C 1mM PBA Eif2α


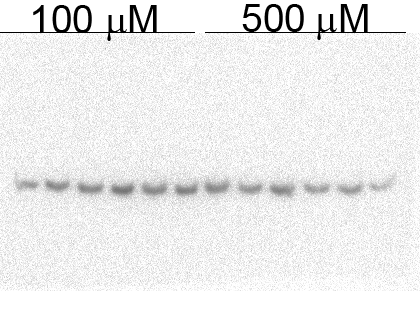


Supplemental Fig. 5D 100µM and 500µM PBA phospho-Eif2α


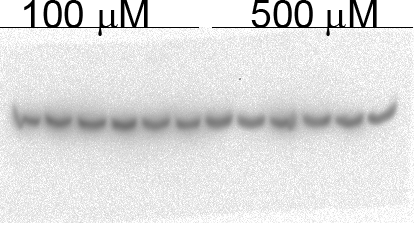


Supplemental Fig. 5D 100µM and 500µM PBA Eif2α


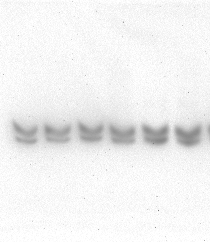


Supplemental Fig. 5D 1mM PBA phospho-Eif2α


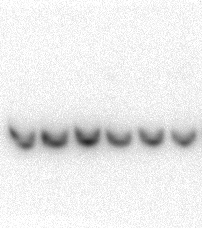


Supplemental Fig. 5D 1mM PBA Eif2α


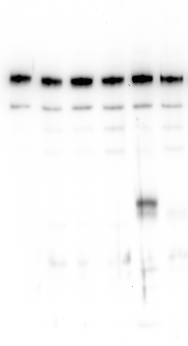


Supplemental Fig. 6A intracellular Collagen III


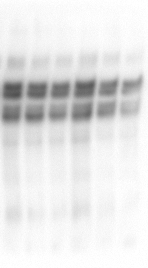


Supplemental Figure 6B extracellular collagen III G189S 5mM PBA


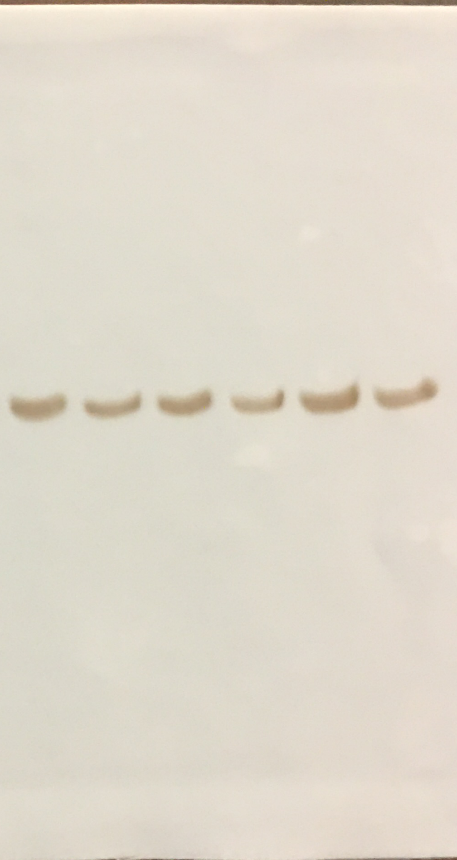


Supplemental Figure 6B tubulin G189S 5mM PBA


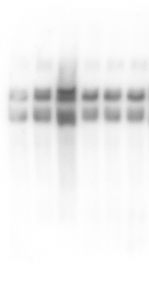


Supplemental Figure 6B extracellular collagen III 500µM PBA 72 hours (top band)


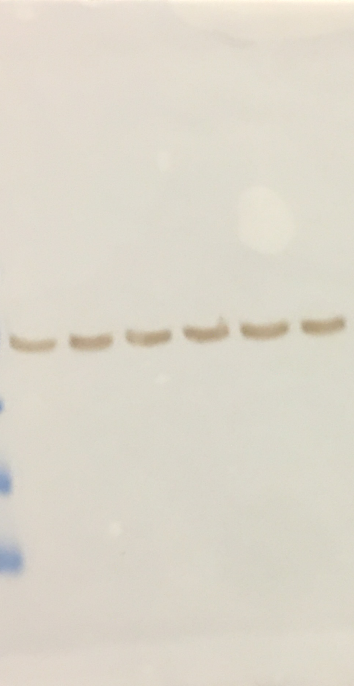


Supplemental Figure 6B tubulin 500µM PBA 72 hours
